# Two Anti-inflammatory Steroidal Saponins from *Dracaena angustifolia* Roxb

**DOI:** 10.3390/molecules18088752

**Published:** 2013-07-24

**Authors:** Hui-Chi Huang, Ming-Kuem Lin, Syh-Yuan Hwang, Tsong-Long Hwang, Yao-Haur Kuo, Chi-I Chang, Chung-Yi Ou, Yueh-Hsiung Kuo

**Affiliations:** 1 Department of Chinese Pharmaceutical Sciences and Chinese Medicine Resources, China Medical University, Taichung 404, Taiwan; 2 Endemic Species Research Institute, Council of Agriculture, Nantou 552, Taiwan; 3 Graduate Institute of Natural Products, Chang Gung University, Taoyuan 333, Taiwan; 4 Division of Herbal Drugs and Natural Products, National Research Institute of Chinese Medicine, Taipei 211, Taiwan; 5 Graduate Institute of Integrated Medicine, College of Chinese Medicine, China Medical University, Taichung 404, Taiwan; 6 Department of Biological Science and Technology, National Pingtung University of Science and Technology, Pingtung 912, Taiwan; 7 Graduate Institute of Pharmaceutical Chemistry, China Medical University, Taichung 404, Taiwan; 8 Tsuzuki Institute for Traditional Medicine, College of Pharmacy, China Medical University, Taichung 404, Taiwan

**Keywords:** Agavaceae, *Dracaena angustifolia* Roxb, steroidal saponins, drangustosides A–B, anti-inflammatory activity

## Abstract

Two new steroidal saponins, named drangustosides A–B (**1**–**2**), together with eight known compounds **3**–**10** were isolated and characterized from the MeOH extract of *Dracaena angustifolia* Roxb. The structures of compounds were assigned based on 1D and 2D NMR spectroscopic analyses, including HMQC, HMBC, and NOESY. Compounds **1** and **2** showed anti-inflammatory activity by superoxide generation and elastase release by human neutrophils in response to fMLP/CB.

## 1. Introduction

The genus *Dracaena* (Agavaceae) includes more than 50 species found in tropical and subtropical regions of the eastern hemisphere. *Dracaena angustifolia* Roxb. is a native shrub or small tree in southern Taiwan, widely planted in Australia, India, Malaysia, and Philippines [1]. The decoction of the underground parts of this plant is used as a tonic and for the treatment of asthma, diarrhea, and inflammation [2]. Previous phytochemical investigations on the genus *Dracaena* have reported the presence of a variety of components, including steroidal saponins [3,4,5,6,7], flavonoids [5,8,9,10], and phenolic compounds [8,11]. The pharmacological investigation indicated that the C_27_ steroidal saponins present on the genus *Dracaena* showed broad biological activities, such as anti-inflammatory, antifungal, antimicrobial, antiviral, analgesic, antioxidative, cytotoxic, and hypoglycemic properties [3,4,5,11]. *D. angustifolia* Roxb. has been studied, and this revealed the presence of several steroidal saponins with antifungal [4], antituberculosis [12], antiproferative [7], and cytotoxic activities [13]. In our continuing phytochemical investigation on *D. angustifolia* Roxb., we have now further identified two new steroidal saponins, drangustosides A–B (**1**–**2**), one known steroidal saponin, alliospiroside A (**3**) [14], and seven benzenoids **4**–**10** in the MeOH extract of *D.angustifolia* Roxb., and report herein the structural determination of these substances using extensive spectroscopic methods. Neutrophils play a significant role in the pathogenesis of several inflammatory diseases. The production of vast amounts of superoxide anion and elastase by activated neutrophils can cause tissue damage and contribute to the development of a wide spectrum of airway inflammatory diseases [15,16]. The anti-inflammatory activity of new compounds was also evaluated as inhibitory activities against formyl-L-methionyl-L-leucyl-L-phenylalanine (fMLP)-induced superoxide anion production and elastase release in human neutrophils. 

## 2. Results and Discussion

**Figure 1:**
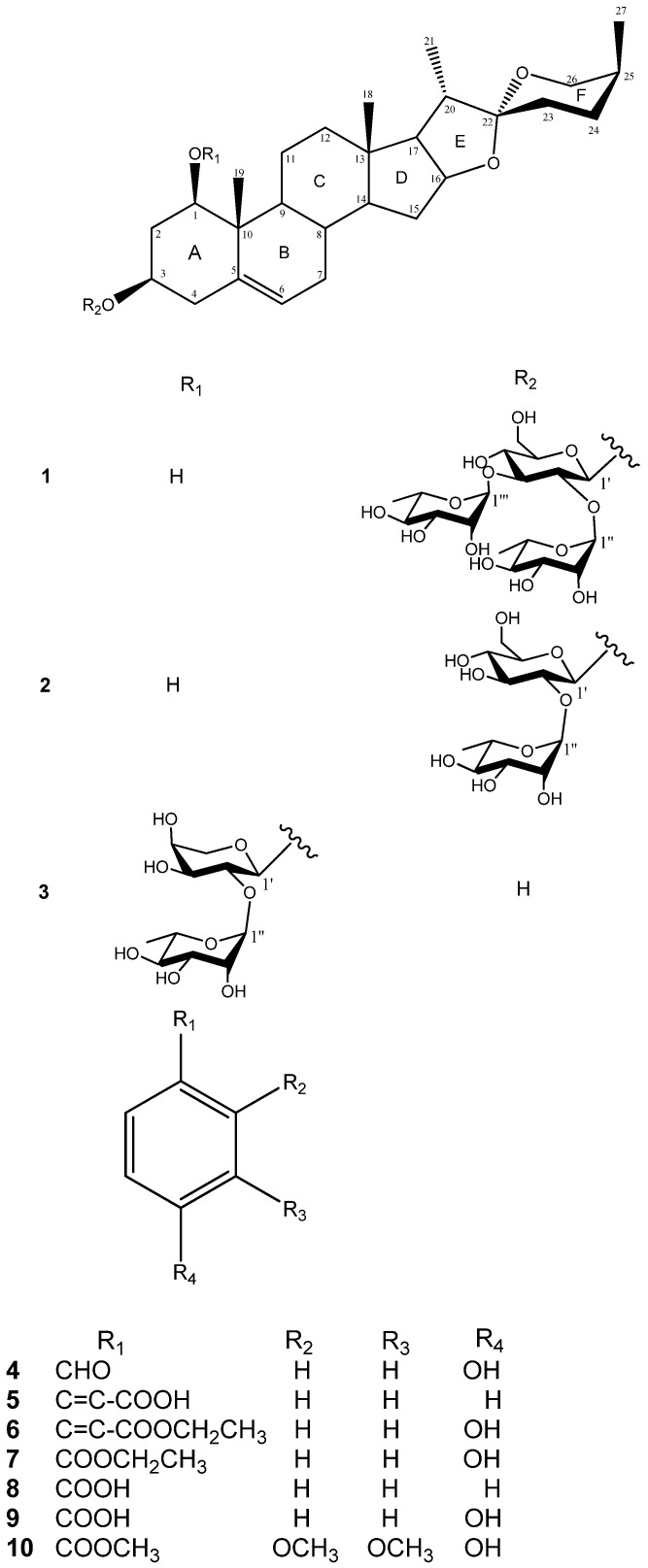
Chemical structure of compounds **1**–**10** from *D. angustifolia* Roxb.

The MeOH extracts of the whole plant of *D. angustifolia* Roxb. was extracted successively with EtOAc and *n*-BuOH. Compounds **1**–**10** (Figure 1) were obtained from the EtOAc fraction by using a series of chromatographic techniques on silica gel, Sephadex LH-20, and RP-HPLC. The structures of the two new steroidal saponins **1**–**2** were elucidated as follows: 

Compound **1** was obtained as a white amorphous solid. Its molecular formula was established as C_45_H_72_O_17_ based on an [M+Na]^+^ ion peak at *m*/*z* 907.4667 in its HRESIMS. The IR spectrum of **1** indicated the existence of hydroxyl groups (3,389) and the characteristic absorption bands of a (25*S*)-spiroketal at 987, 918, 898, and 843 (intensity 918 > 898) cm^−1^ [14]. The ^1^H-NMR spectrum (Table 1) of **1** showed signals due to two tertiary methyl groups (*δ*_H_ 1.34 and 0.87), four secondary methyl groups [*δ*_H_ 1.06 (d, *J* = 7.0 Hz), 1.08 (d, *J* = 7.0 Hz), 1.62 (d, *J* = 6.2 Hz), and 1.68 (d, *J* = 6.2 Hz)], one olefinic proton [*δ*_H_ 5.53 (d, *J* = 5.1 Hz)], and three monosaccharide anomeric protons [*δ*_H_ 4.91 (d, *J* = 7.7 Hz), 5.73 (br s), and 5.82 (br s)], two of which were considered to be L-rhamnosides. Based on the chemical shifts and coupling constants, the three monosaccharides were considered as a combination of one D-glucoside and two L-rhamnosides. The ^13^C-NMR spectrum (Table 1) exhibited 45 carbon atoms, 27 of which belonged to the aglycone carbons while the remaining were due to three hexose sugar units. The ^13^C-NMR and DEPT spectra revealed C_27_ signals including four methyl, nine methylene, ten methine, and four quaternary carbons. Among the four quaternary carbon signals, the signal at δ_C_ 110.0 was identified as an acetal carbon (C-22) and the signal at δ_C_ 139.3 was assigned as a diakyl substituted olefinic carbon (C-5). The aforementioned data suggested that **1** is possibly a spirostanol glycoside with the aglycone being a 27-carbons skeletonal aglycone along with three sugar moieties [4]. 

**Table 1:** ^1^H-NMR and ^13^C-NMR spectroscopic data (in ppm, *J* in Hz) of compounds **1**–**3** in CD_3_OD.

	1 *δ*_H_	*δ*_C_		2 *δ*_H_	*δ*_C_		3 *δ*_H_	*δ*_C_
1	3.68 (dd, 11.6, 3.9)	78.1		3.70 (dd, 12.4, 4.2)	78.2		3.86 (dd, 12.1, 3.7)	83.9
2	2.58 (br d, 12.5),	41.9		2.63 (br d, 12.4),	41.1		2.72 (br d, 12.4),	37.7
	2.28 (q, 11.8)			2.36 (q, 11.9)			2.36 (q, 11.8)	
3	4.02 (m)	75.0		4.06 (m)	75.1		3.86 (m)	68.5
4	2.77 (m), 2.75 (m)	39.4		2.84 (m), 2.82 (m)	39.7		2.61, 2.57	44.1
5	-	139.3		-	139.4		-	139.9
6	5.53 (d, 5.1)	125.5		5.54 (d, 6.2)	125.4		5.57 (d, 5.2)	125.0
7	1.90 (m), 1.54 (br d, 9.6)	32.7		1.92 (m), 1.57 (m)	32.6		1.89 (m), 1.55 (m)	32.3
8	1.58 (m)	33.2		1.59 (m)	33.2		1.57 (m)	33.4
9	1.32 (m)	51.4		1.36 (m)	51.5		1.50 (m)	50.7
10	-	44.0		-	44.1		-	43.2
11	2.83 (dd, 11.3, 3.5), 1.70 (m)	24.4		2.85 (m)	24.4		2.91 (m)	24.3
				1.75 (m)			1.63 (m)	
12	1.72 (m), 1.21 (m)	40.7		1.73 (m), 1.23 (m)	40.8		1.54 (m), 1.28 (m)	40.6
13	-	40.4		-	40.5		-	40.5
14	1.13 (m)	57.1		1.13 (m)	57.1		1.13 (m)	57.1
15	2.05 (m), 1.47 (m)	32.5		2.07 (m), 1.49 (m)	32.7		2.01 (m), 1.39 (m)	32.7
16	4.51 (dd, 14.5, 7.9)	81.6		4.51 (dd, 14.7, 8.0)	81.4		4.50 (dd, 14.5, 7.5)	81.5
17	1.77 (t, 7.9)	63.2		1.78 (t, 8.0)	63.3		1.71 (t, 7.5)	63.1
18	0.87 (s)	16.8		0.90 (s)	16.8		0.84 (s)	17.0
19	1.34 (s)	14.0		1.37 (s)	14.0		1.44 (s)	15.3
20	1.87 (m)	42.7		1.88 (m)	42.8		1.84 (m)	42.7
21	1.08 (d, 7.0)	15.1		1.11 (d, 6.9)	15.1		1.10 (d, 7.0)	15.1
22	-	110.0		-	110.0		-	110.0
23	1.88 (m), 1.43 (m)	26.7		1.91 (m), 1.45 (m)	26.7		1.86 (m), 1.40 (m)	26.7
24	2.11 (m), 1.36 (m)	26.5		2.15 (m), 1.37 (m)	26.5		2.11 (m), 1.34 (m)	26.5
25	1.58 (m)	27.8		1.61 (m)	27.8		1.55 (m)	27.8
26	4.05 (br d, 11.5),	65.3		4.09 (br d, 10.8),	65.3		4.06 (br d, 10.8),	65.3
	3.34 (d, 11.5)			3.36 (d, 10.8)			3.35 (d, 10.8)	
27	1.06 (d, 7.0)	16.5		1.07 (d, 7.0)	16.6		1.06 (d, 7.1)	16.6
	3-*O*-β-D-Glc			3-*O*-β-D-Glc			3-*O*-α-L-Ara	
1'	4.91 (d, 7.7)	100.2		5.06 (d, 7.3)	100.7		4.72 d (7.0)	100.7
2'	3.99 (dd, 8.0, 7.7)	78.3		4.23 (m)	78.0		4.59 (m)	75.4
3'	4.14 (t, 8.0)	87.6		4.26 (m)	79.9		4.08 (m)	76.2
4'	4.03 (m)	70.2		4.16 (t, 8.6)	72.9		4.06 (m)	70.4
5'	3.78 (m)	78.3		3.88 (m)	78.5		4.25 (m)3.66 (d, 11.0)	67.6
6'	4.38 (br d, 11.5),	62.4		4.46 (dd, 11.8, 1.9),	62.8		-	
	4.31 (dd, 11.5, 5.9)			4.31 (m)				
	α-L-RhaI			α-L-Rha			α-L-Rha	
1''	5.82 (br s)	104.1		6.36 (br s)	102.3		6.31 (br s)	101.9
2''	4.73 (m)	72.9		4.80 (m)	72.8		4.70 (br d, 3.5)	72.9
3''	4.52 (m)	73.0		4.64 (dd, 9.2, 3.1)	73.1		4.63 (dd, 9.3, 3.5)	72.8
4''	4.46 (m)	74.0		4.34 (m)	74.4		4.31 (dd, 9.3, 9.3)	74.8
5''	4.76 (dq, 8.9, 6.2)	70.1		4.99 (m)	69.7		4.85 (dq, 9.3, 6.2)	69.5
6''	1.62 (d, 6.2)	18.6		1.71 (d, 6.2)	18.9		1.73 (d, 6.2)	19.3
	α-L-RhaII							
1'''	5.73 (br s)	102.8						
2'''	4.81 (br s)	72.7						
3'''	4.46 (m)	72.8						
4'''	4.29 (m)	73.8						
5'''	4.86 (dq, 9.1, 6.2)	70.9						
6'''	1.68 (d, 6.2)	18.9						

**Figure 2:**
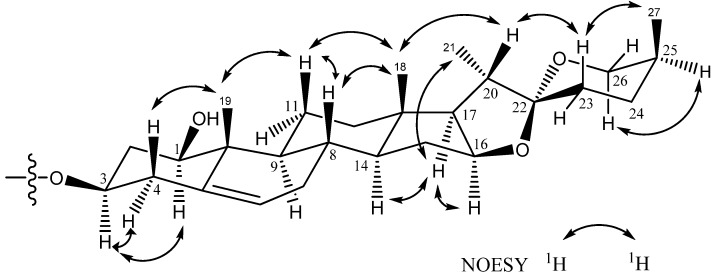
Key NOESY correlations of the aglycone moiety of **1**–**2**.

Comparison of the ^1^H and ^13^C-NMR signals (Table 1) of the aglycone moiety of **1** with those of alliospiroside A (**3**) [14], indicated that the structures of the aglycone parts of **1** and **3** were almost superimposable. The only significant differences were seen in the ^13^C-NMR signals of C-1 and C-3 in the ring A portion. Generally, a carbon attached to an -*O*-glycoside group exhibits the higher ^13^C NMR shift than that of a carbon attached to a hydroxyl group. The *O*-glycoside group in compound **3** was assigned at C-1 [*δ*_C__-1_ 83.9; *δ*_H-1_ 3.86 (dd, *J* = 12.1, 3.7 Hz) and *δ*_C__-3_ 68.5, *δ*_H-3_ 3.86 (m)]. The NMR peaks of C-1 were at *δ*_C_ 78.1 [*δ*_H-1_ 3.68 (dd, *J* = 11.6, 3.9 Hz)] and C-3 at *δ*_C_ 75.0 [*δ*_H-3_ 4.02 (m)] were in compound **1**, while the anomeric proton H-1′ [*δ*_H_ 4.91 (d, *J* = 7.7 Hz)] exhibited an HMBC correlation with *δ*_C_ 75.0 (C-3). These results lead to the conclusion that the -*O*-glycoside is unambiguously located at C-3 in compound **1**. Comparing the NMR data of C-1 and C-3 between **1** and **3**, the -*O*-glycosidation at C-3 in compound **1** and at C-3 in compound **3** agrees with the general knowledge. The coupling constant of H-1 (dd, *J* = 11.6, 3.9 Hz) can be assigned to an α-axial orientation. H-1 and H-3 have a NOESY correlation, therefore revealing that H-3 was located with the same α-axial orientation. In general, the difference in chemical shifts between axial and equatorial protons among on H_2_-23, H_2_-24, and H_2_-26 can be used to resolve the absolute configuration of C-25 [17,18]. The difference in chemical shift (Δea = *δ*e − *δ*a) for H_2_-23, -24, -26 are usually > 0.35 for 25*S* configurations whereas it is < 0.20 ppm in 25*R* compounds. Therefore, the axially oriented H_3_-27 (1.06) and *S*-configuration of C-25 were deduced based on the presence of the following different chemical shift data: Δea = 0.45 for H_2_-23, Δea = 0.75 for H_2_-24, Δea = 0.71 for H_2_-26. The 22*R* stereochemistry was deduced based on the presence of the two proton signals at the C-26 of spirostanol appeared as separate signals at *δ*_H_4.05 (H-26eq) and 3.34 (H-26ax)] and the spirostanol had a normal type F ring (Figure 1) [18]. The correlations from H-26ax (*δ*_H_ 3.34) to H-25 (*δ*_H_ 1.58) and from H-23ax (*δ*_H_ 1.43) to H-20 (*δ*_H_ 1.87) and H_3_-27 (*δ*_H_ 1.06) in the NOESY spectrum (Figure 2) were consistent with the C-22*R* and C-25*S* configurations. The stereostructure of 1 was confirmed by NOESY analysis, as shown in Figure 2. 

**Figure 3:**
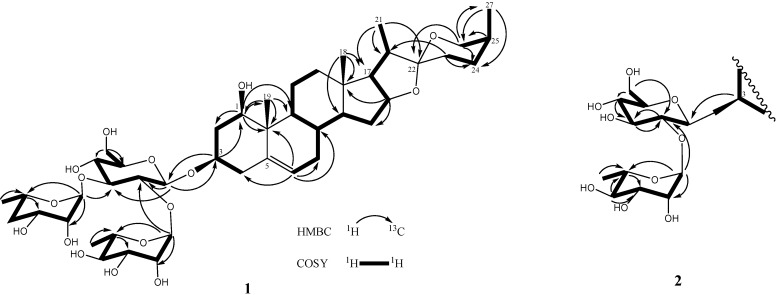
The COSY and key HMBC correlations of compounds **1**–**2**.

D-Glucose and L-rhamnose in a 1:2 ratio were obtained upon acidic hydrolysis of **1** with HCl in 1,4-dioxane [19], as indicated by chiral HPLC methodology [20]. Analysis of the NMR data indicated the presence of a trisaccharide unit connected to an aglycone moiety, with three anomeric protons [*δ*_H_ 4.91 (d, *J* = 7.7 Hz, H-1' of *β*-D-glucopyranosyl), 5.73 (br s, H-1''' of *α*-L-rhamnopyranosyl), and 5.82 (br s, H-1'' of *α*-L-rhamnopyranosyl)] which correlated to carbon signals at *δ*_C_ 100.2, 102.8, and 104.1, respectively [21]. In the TOCSY spectrum of **1**, the anomeric proton that was ascribed to D-glucoside. [*δ*_H_ 4.91 (d, *J* = 7.7 Hz, H-1')] showed connectivity with four consecutive methines as follows: *δ*_H_ 3.99 (H-2'), 4.14 (H-3'), 4.03 (H-4'), and 3.78 (H-5'), and then two methylene protons [*δ*_H_ 4.38 (H-6a'), and 4.31 (H-6b′)]. The TOCSY spectrum gave a result that showed two rhamnoside units connected on the glucoside unit. In the HMBC spectrum, a ^3^*J* correlation between H-1'' (*δ*_H_ 5.82) of rhamnoside and C-2' (*δ*_C_ 78.3) of glucoside, and between H-1''' (*δ*_H_ 5.73) of rhamnoside and C-3' (*δ*_C_ 87.6) of glucoside confirmed that two rhamnoside units located at C-2' and C-3' of glucoside units, respectively (Figure 3). Consequently, the connectivity of the three monosaccharide in **1** was identified as 3-*O*-{*α*-L-rhamnopyranoside(1→2)-*O*-[*α*-L-rhamnopyranoside(1→3)]-β-D-glucopyranosyl}. Therefore, the structure **1** was ultimately identified as 3-*O*-{*α*-L-rhamnopyranoside(1→2)-*O*-[*α*-L-rhamno-pyranoside(1→3)]-*β*-D-glucopyranosyl} (1,3,22*R*,25*S*)-spirost-5-ene-1*β*,3*β*-diol, to which the trivial name drangustoside A was given.

Compound **2** was obtained as an amorphous solid with a molecular formula of C_39_H_62_O_13_ determined by the HRESIMS data (*m/z* 761.4085 [M+Na]^+^). The IR spectrum of **2** indicated the existence of hydroxyl groups (3,389) and the characteristic absorption bands of (25*S*)-spiroketal at 987, 920, 897, and 840 (intensity 920 > 897) cm^−1^ [14]. The ^1^H- and ^13^C-NMR spectroscopic features of the aglycone moiety of **2** (Table 1) were very similar to those of **1**, which was suggested that compounds **2** and **1** possessed the same (1,3,22*R*,25*S*)-spirost-5-ene-1,3-diol aglycone. The ^1^H- and ^13^C-NMR spectra of **2** exhibited two anomeric protons signals at *δ*_H_ 6.36 (br s) and 5.06 (d, *J* = 7.3 Hz), as well as the corresponding anomeric carbon resonances at *δ*_C_ 102.3 and 100.7, respectively, that established the existence of two sugar units. The differences in the ^1^H and ^13^C-NMR spectra of **1** and **2** showed that **2** had one less rhamnoside unit than **1**. In addition, the molecular ion mass spectrum of **2** was 146 atomic units lower than that of **1**, and only two anomeric proton signals were observed at *δ*_H_ 5.06 (d, *J* = 7.3 Hz) and 6.36 (br s). Acid hydrolysis of **2** yielded a L-rhamnose and a D-glucose in 1:1 ratio. The TOCSY spectrum of **2** showed that the proton at *δ*_H_ 5.06 (H-1′) was coupled to the signals at *δ*_H_ 4.23 (H-2′), 4.26 (H-3′), 4.16 (H-4′), 3.88 (H-5′), 4.31 (H-6′) and 4.46 (H-6′), successively. The carbon signals at *δ*_C_ 100.7, 78.0, 79.9, 72.9, 78.5, and 62.8 were assigned to the D-glucoside C-1′, C-2′, C-3′, C-4′, C-5′, and C-6′, respectively, from the HSQC analysis. The proton and carbon signals of L-rhamnoside were fully assigned by the same method. Moreover, the α-anomeric configurations of the L-rhamnoside units [*δ*_H_ 6.36 (br s)] and the β-anomeric configurations of the D-glucoside units [*δ*_H_ 5.06 (d, *J* = 7.3 Hz)] were confirmed by their coupling constants and chemical shifts [19]. In the HMBC spectrum, ^3^*J*-correlations were observed between H-1′′ (δ_H_ 6.36) of rhamnoside and C-2′ (δ_C_ 78.0) of glucoside and H-1′ (δ_H_ 5.06) of glucoside and C-3 (δ_C_ 75.1) of aglycone. These findings indicated that the terminal rhamnose was linked at C-2′ of the inner glucose and the sugar chain was attached to C-3 of the aglycone (Figure 3). Consequently, compound **2** was identified as (1,3,22*R*,25*S*)-spirost-5-ene-1,3-diol 3-*O*-[*α*-L-rhamnopyranoside (1→2)-*β*-D-glucopyranosyl] and named drangustoside B. 

The other eight isolated compounds were identified as alliospiroside A (**3**) (ESIMS *m*/*z* 709 [M+H]^+^, C_38_H_60_O_12_) [14], *p*-hydroxybenzaldehyde (**4**) (ESIMS *m*/*z* 123 [M+H]^+^, C_7_H_6_O_2_) [22], (*E*)-cinnamic acid (**5**) (ESIMS *m*/*z* 149 [M+H]^+^, C_9_H_8_O_2_) [23], ethyl (*E*)-4-hydroxycinnamate (**6**) (ESIMS *m*/*z* 193 [M+H]^+^, C_11_H_12_O_3_) [24], ethyl 4-hydroxybenzoate (**7**) (ESIMS *m*/*z* 167 [M+H]^+^, C_9_H_10_O_3_) [25], benzoic acid (**8**) (ESIMS *m*/*z* 123 [M+H]^+^, C_7_H_6_O_2_) [26], 4-hydroxybenzoic acid (**9**) (ESIMS *m*/*z* 139 [M+H]^+^, C_7_H_6_O_3_,) [27], and methyl 2,3-dimethoxy-4-hydroxybenzoate (**10**) (ESIMS *m*/*z* 213 [M+H]^+^, C_10_H_12_O_5_) [28], respectively, by comparison with the spectroscopic data reported in the literature for these compounds. 

Inhibition of superoxide generation and elastase release by human neutrophils in response to fMLP were utilized to measure the anti-inflammatory activity of compounds **1** and **2** (Table 2). LY294002, a phosphatidylinositol-3-kinase inhibitior, was used as a positive control for inhibition of superoxide anion generation and elastase release with IC_50_ values of 2.00 ± 0.59 and 4.94 ± 1.69 μM, respectively. Compound **1** showed the highest anti-inflammatory activity against superoxide generation (IC_50_= 18.55 ± 0.23 μM) and elastase (IC_50_= 1.74 ± 0.25 μM). 

**Table 2:** Inhibitory effects of pure compounds on superoxide generation and elastase release in fMLP/CB-induced human neutrophils.

Compound	Superoxide anion IC_50_ (μM) ^a^	Elastase release IC_50_ (μM) ^a^
1	26.39 ± 1.63	3.94 ± 0.19
2	18.55 ± 0.23	1.74 ± 0.25
LY294002	2.00 ± 0.59	4.94 ± 1.69

^a^ Concentration necessary for 50% inhibition (IC_50_). Results are presented as mean ± S.E.M. (n = 2 or 3).

## 3. Experimental

### 3.1. General Procedures

The infrared (IR) spectra were measured on a Mattson Genesis II spectrophotometer using a KBr matrix. The optical rotations were measured on a JASCO P-1020 polarimeter equipped with a sodium lamp (589 nm). ^1^H- and ^13^C-NMR spectra were recorded on a Bruker DRX-500 spectrometer with CD_3_OD as the solvent. The HRESIMS data were collected using a Finnigan MAT95S mass spectrometer. The GC-MS was performed using a Thermo Finnigan TRACE GC Ultra instrument. Sephadex LH-20, and silica gel (Merck 70–230 mesh and 230–400 mesh) were used for the column chromatography. The preparative HPLC was performed using a reverse phase column (Cosmosil 5C_18_-AR-II column, 5 μm particle size, 250 mm × 20 mm i.d.) and the stereoselective HPLC analysis was performed using a normal phase column (Chiralpak AD-H column, 5 μm particle size, 250 mm× 10 mm i.d.) on a Shimadzu LC-6AD series apparatus with a RID-10A Refractive Index detector. The MPLC was performed using a reverse phase column (Buchi MPLC glass column, C_18_, 460 mm × 36 mm i.d.) on a Buchi pump module C-601 series apparatus without detector.

### 3.2. Plant Material

The leaves of *D. angustifolia* Roxb. used in this experiment was collected on the mountains of Nantou County, Taiwan, in September 2009. The plant was identified by Dr. Shy-Yuan Hwang, Endemic Species Research Institute. A voucher specimen of *D. angustifolia* Roxb (No. CMR200909DA) was deposited in the Department of Chinese Pharmaceutical Sciences and Chinese Medicine Resources, China Medical University, Taichung, Taiwan.

### 3.3. Extraction and Isolation

The leaves of *D. angustifolia* Roxb. (6.5 kg) were sectioned and extracted five times with MeOH (20 L) for 72 hr each time. The MeOH extract was continuously dried under reduced pressure at 45 °C to yield the brown syrup (*ca*. 972.4 g). The combined extracts were suspended on H_2_O (3 L) and then successively partitioned with EtOAc (3 L, three times) and *n*-BuOH (3 L, three times). The EtOAc layer (57.7 g) was subjected to silica gel CC (6.5 × 43.5 cm, Merk 70–230 mesh), eluting with a CHCl_3_/MeOH gradient to give ten fractions (fr. 1–10). Fraction 3 (679.2 mg) was again subjected to Sephadex LH-20 column chromatography eluting with MeOH to yield five subfractions (fr. 3.1–3.5). Fr. 3.3 (141.8 mg) was subjected to silica gel CC (3 × 25 cm, Merk 70–230 mesh) with gradient *n*-hexane-EtOAc to yield nine subfractions (fr. 3.3.1–3.3.9). Fr. 3.3.5 was chromatographed using preparative RP-HPLC (Cosmosil 5C_18_-AR-II column, flow rate: 5.0 min/mL) with isocratic MeOH-H_2_O (65:35) to yield **7** (6.0 mg), **8** (3.6 mg), and **10** (3.5 mg). Using preparative RP-HPLC (Cosmosil 5C_18_-AR-II column, flow rate: 5.0 min/mL) with isocratic MeOH-H_2_O (60:40), **4** (8.4 mg), **5** (4.6 mg), **6** (5.5 mg), and **9** (2.1 mg) were obtained from fr. 3.3.7. Fraction 7 was subjected to Sephadex LH-20 column (10 × 70 cm) chromatography eluting with MeOH to yield six subfractions (fr.7.1–7.6). Fr. 7.4 was subjected to preparative RP-HPLC (Cosmosil 5C_18_-AR-II column, flow rate: 5.0 min/mL), eluting with MeOH-H_2_O (55:45) to yield **2** (3.6 mg). Fr. 8 was further separated by chromatography on RP-MPLC (Buchi MPLC glass column, flow rate: 8.0 min/mL) with gradient MeOH/H_2_O (0%-100%) to yield 14 subfractions (fr.8.1–8.14). Fraction 8.4 was further separated by chromatography on Sephadex LH-20 (10 × 70 cm) with MeOH to yield six subfractions (fr. 8.4.1–8.4.6). Fr. 8.4.4 was chromatographed using RP-HPLC with isocratic MeOH-H_2_O (70:30) to yield **1** (5.7 mg) and **3** (5.4 mg).

### 3.4. Spectral Data

*Drangustoside A* (**1**). white amorphous powder; [α]^25^_D_ −54.74 (*c* 0.29, MeOH); IR (KBr) *ν*_max_ 3,389 (OH), 2,928, 1,681 (C=C), 1,367, 1,043 (C-O-C), 987, 918, 898 and 843 (intensity 918 > 898, spiroketal chain of the 25*S* series) cm^-1^; for ^1^H- and ^13^C-NMR spectroscopic data, see Table 1; HRESIMS *m/z* 907.4667 [M+Na]^+^ (calcd for C_45_H_72_O_17_Na, 907.4662). 

*Drangustoside B* (**2**). white amorphous powder; [α]^25^_D_ −38.25 (*c* 0.15, MeOH); IR (KBr) *ν*_max_ 3,389 (OH), 2,929, 1,681 (C=C), 1,368, 1,044 (C-O-C), 987, 920, 897 and 840 (intensity 920 > 897, spiroketal chain of the 25*S* series) cm^−1^; for ^1^H- and ^13^C-NMR spectroscopic data, see Table 1; HRESIMS *m/z* 761.4085 [M+Na]^+^ (calcd for C_39_H_62_O_13_ Na, 761.4088).

### 3.5. Acid Hydrolysis of **1**–**2**

Compound **1** or **2** (2 mg each) were heated with 1N HCl (dioxane-H_2_O, 1:1, 2 mL) at 90 °C for 4 h, and then evaporated under reduced pressure to give a residue. The residue was partitioned with CH_2_Cl_2_and H_2_O three times. The aqueous (pH = 2.0) was neutralized with aqueous 1N NaOH and then evaporated under reduced pressure. The aqueous layer (0.3 mg; pH = 7.0) was dissolved in MeOH (0.5 mL) and then analyzed by stereoselective HPLC under the following conditions: column: Chiralpak AD-H; solvent system: *n*-hexane-ethanol-TFA (7:3:0.1, v/v); flow rate: 0.5 mL/min; inject volume: 20 μL; detector: refractive index. D-Glucose and L-rhamnose for compounds **1** (t_R_= 14.31 and 15.80 min, respectively) and **2** (t_R_ = 14.36 and 15.89 min, respectively) were determined by comparison of the respective retention times of D-glucose (t_R_= 14.33 min) and L-rhmanose (t_R_ =15.85 min) standards. 

### 3.6. Superoxide Generation and Elastase Release by Human Neutrophils

Human neutrophils were obtained using dextran sedimentation and Ficoll centrifugation. Blood was drawn from healthy human donors (20–32 years old) by venipuncture using a protocol approved by the institutional review board at Chang Gung Memorial Hospital. Neutrophils were isolated using a standard method as previously described [29,30]. Measurements of superoxide anion generation and elastase release were carried out according to previously described procedures [29,30].

## 4. Conclusions

Two new steroidal saponins **1**–**2**, one known steroidal saponin **3**, and seven known benzenoids **4**–**10** were isolated from the MeOH extract of *D. angustifolia* Roxb. Their structures were determined based on extensive spectroscopic analyses, and the anti-inflammatory activity of **1** and **2** were evaluated by superoxide anion generation and elastase assays, which revealed that the steroidal saponin (1,3,22*R*,25*S*)-spirost-5-ene-1,3-diol with a disaccharide, **2**, had better anti-inflammatory activity against superoxide generation than **1** with a trisaccharide. Another reported steroidal saponin with a disaccharide, 3-*O*-β-chacotriosyl-25(*S*)-spirost-5-en-3β-ol [21], which was similar to **2**, but missing an hydroxyl group at C-1, also showed very potent anti-inflammatory activity against superoxide generation. These results implied that the number of sugars in this type of steroidal saponins could be important for the anti-inflammatory activity. Moreover, 3-*O*-β-chacotriosyl-25(*S*)-spirost-5-en-3-ol (IC_50_ = 4.65 ± 0.25 μM, reported data) had more potent anti-inflammatory activity against superoxide generation than compound **2** (IC_50_ = 18.55 ± 0.23 μM); whereas, **2** (IC_50_ = 1.74 ± 0.25 μM) was more active than 3-*O*-β-chacotriosyl-25(*S*)-spirost-5-en-3β-ol (IC_50_ = 4.65 ± 0.25 μM, reported data) in the human neutrophil elastase release assay [21]. Thus, the functional group in C-1 for this type of steroidal saponins seems to play a crucial role for the anti-inflammatory activity against superoxide generation and human neutrophil elastase release.
